# Role of Lipid Rafts in Pathogen-Host Interaction - A Mini Review

**DOI:** 10.3389/fimmu.2021.815020

**Published:** 2022-01-20

**Authors:** Rakesh Kulkarni, Erik A. C. Wiemer, Wen Chang

**Affiliations:** ^1^ Molecular and Cell Biology, Taiwan International Graduate Program, National Defense Medical Center, Academia Sinica and Graduate Institute of Life Science, Taipei, Taiwan; ^2^ Institute of Molecular Biology, Academia Sinica, Taipei, Taiwan; ^3^ Medical Oncology, Erasmus MC Cancer Institute, University Medical Center, Rotterdam, Netherlands

**Keywords:** lipid rafts, vaccinia virus, major vault protein, pathogen-host interactions, cell entry

## Abstract

Lipid rafts, also known as microdomains, are important components of cell membranes and are enriched in cholesterol, glycophospholipids and receptors. They are involved in various essential cellular processes, including endocytosis, exocytosis and cellular signaling. Receptors are concentrated at lipid rafts, through which cellular signaling can be transmitted. Pathogens exploit these signaling mechanisms to enter cells, proliferate and egress. However, lipid rafts also play an important role in initiating antimicrobial responses by sensing pathogens *via* clustered pathogen-sensing receptors and triggering downstream signaling events such as programmed cell death or cytokine production for pathogen clearance. In this review, we discuss how both host and pathogens use lipid rafts and associated proteins in an arms race to survive. Special attention is given to the involvement of the major vault protein, the main constituent of a ribonucleoprotein complex, which is enriched in lipid rafts upon infection with vaccinia virus.

## Introduction

The fluid mosaic model of biological membranes was proposed by Singer and Nicolson in 1972, whereby membranes are composed of uniform lipid bilayers in which select proteins randomly float (1). Later studies have contradicted this hypothesis, revealing instead the presence of detergent-resistant and detergent-soluble fractions in cell membranes (2). This latter heterogeneity in cell membranes was identified to be due to the presence of lipid rafts or microdomains (3–5). Lipid rafts are small, dynamic, heterogeneous microdomains (10-200 nm) that are enriched in cholesterol and glycophospholipids (6–9). High concentrations of sphingolipids and dense packing of protein with cholesterol in lipid rafts promotes cell membrane stability (10). Lipid rafts also contain a diverse group of cellular receptors (11–15) which play important roles in various cellular processes such as endocytosis, exocytosis, receptor trafficking and cell signaling (4). However, these same lipid rafts are also exploited by many pathogens to achieve cell entry and cell exit e.g. *via* budding (8, 16).

**Figure 1:**
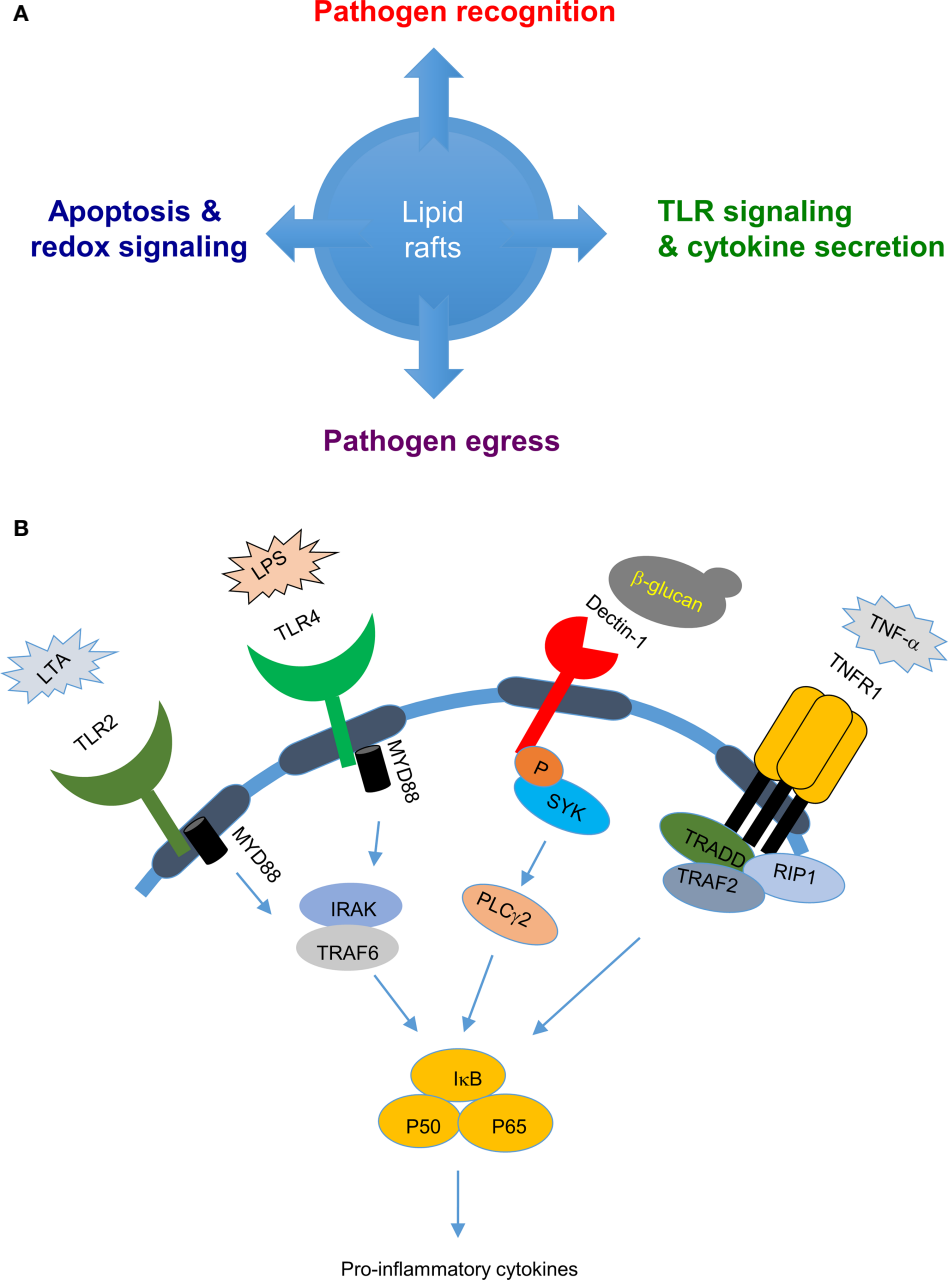
Biological roles of lipid rafts in pathogen-host interactions: **(A)** Lipid rafts are involved in several cellular functions, such as pathogen recognition, cell signaling, and pathogen egress which decides the outcome of pathogen-host interaction. **(B)** Lipid rafts in pathogen sensing and cytokine release: Lipid rafts play an important role in pathogen sensing by recruiting pathogen sensing receptors such as toll like receptors when cells are stimulated with bacterial cell wall components LPS or LTA and C-type lectin receptors when stimulated with fungal cell wall components such as β-glucans which evetually leads to cytokine secretion.

Advancements in cell imaging approaches have allowed lipid rafts to be visualized in cells, with cholera toxin staining frequently used for confocal microscopy-based observations (17). Lipid rafts are relatively resistant to extraction by non-ionic detergents such as 1% Triton X-100 (4, 18, 19), but biochemical enrichment by detergent extraction followed by flotation centrifugation has enabled more detailed analyses of lipid rafts. Many lipid raft-specific markers have been identified, including flotillin and caveolin (12, 15). Important tools to study the impact of lipid rafts in various cell signaling pathways are methyl-β cyclodextrin (MβCD), filipin and nystatin. These compounds are used to extract cholesterol from the plasma membrane and through preferentially targeting cholesterol in lipid rafts, depletes the raft structures (20, 21). Lipid rafts are also found in multiple cells types in brain such as neurons, astrocytes and microglia; several neurodegerative diseases, such as Alzheimer’s, Parkinson’s, Huntington’s, multiple sclerosis and lysosomal storage disease were found to be associated with altered composition of lipid rafts (22–26). Overall, lipid rafts modulate multiple aspects of cellular functions that are important for cell survival, immune signaling as well as pathogen recognition and pathogen egression (**Figure 1A**), as described below.

## Lipid Raft Involvement in Pathogen Recognition Receptor Signaling and Cytokine Secretion

Lipid rafts play important roles in modulating host innate and adaptive immune responses against pathogens. Apart from harboring proteins important for phagocytosis of pathogens (27–29), lipid rafts accumulate pathogen recognition receptors - including c-type lectin receptors (CLRs) and Toll-like receptors (TLRs) - to detect pathogens and initiate downstream signaling cascades for cytokine release and complement system activation for pathogen clearance (30–32) (**Figure 1B**). TLR4 was found to be enriched in lipid raft fractions from cells stimulated with the specific ligand lipopolysaccharide (LPS), but not from non-stimulated cells, and depletion of lipid rafts by nystatin and filipin resulted in failure to produce the downstream cytokine TNF-α; suggesting that lipid rafts are important in TLR4 activation (33). Another TLR, Toll-like receptor 2 (TLR2) that recognizes cell wall components of lipoteichoic acid (LTA) in Gram-positive bacteria, is enriched in lipid rafts and transported to Golgi network upon cell treatment with LTA (34–36). Depletion of lipid rafts by MβCD or nystatin inhibited this enrichment of TLR2 and its transport to Golgi, implying a role for lipid rafts in TLR2 activation and trafficking (34–36). Similarly, host C-type lectins that sense carbohydrate-rich domains on fungi and activate downstream signaling events were also observed to be enriched in lipid rafts (37). Dectin-1 that is primarily expressed on dendritic cells, macrophages and neutrophils plays an important role in anti-fungal immunity (38). Upon sensing fungal zymosan or β-glucan, host dectin-1 was found to translocate with its downstream signaling molecules spleen tyrosine kinase (SYK) and phospholipase C gamma 2 (PLCγ2) to lipid rafts. Depletion of lipid rafts by MβCD treatment resulted in loss of SYK phosphorylation in dendritic cells, supporting a role for lipid rafts in dectin-1 signaling (39). During *Streptococcus pneumoniae* infection, splenic marginal zone (MZ) macrophage lipid rafts accelerate pathogen uptake and degradation (40), as well as mediate DC-SIGN- or SIGN-R1-induced classical complement pathway activation against *S. pneumoniae*, thereby facilitating rapid clearance of this pathogen.

Cytokines are soluble factors released by cells in response to infection and inflammation and they are key modulators of the immune system. Cytokine receptors are recruited to lipid rafts to mediate cytokine signaling. For example, tumor necrosis factor-α receptor 1 (TNFR1) and interferon alpha and beta receptor subunit 1 (IFNAR1) are enriched in lipid rafts, and depletion of lipid rafts reduced cytokine release (41, 42). Furthermore, release of cytokines from vesicles requires N-ethylmaleimide sensitive factor attachment protein receptor (SNARE) mediated fusion with the plasma membrane. SNARE proteins, including syntaxin4 and synaptosomal associated protein-23, are enriched in lipid rafts of LPS-stimulated macrophages, facilitating release of the cytokine TNF-α (43). Other reports also showed that SNARE and Rab proteins are associated with lipid rafts (44, 45).

It is interesting that, although cytokine release is dependent on lipid rafts, the integrity of lipid rafts is also reciprocally affected by cytokine signaling. For example, interferon-induced viperin interacts with farnesyl diphosphate synthase (FPPS) to inhibit cholesterol synthesis and lipid raft formation (46), in addition to its role in catalyzing cytidine triphosphate (CTP) to 3′-deoxy-3′,4′-didehydro-CTP (ddhCTP) (46, 47).

## Role of Lipid Rafts in Apoptosis and Redox Signaling

Reactive oxygen species (ROS) produced in phagosomes eliminate pathogens through oxidative damage by innate immune cells such as neutrophils (48), representing an important element of inflammation and antimicrobial host defense. Nicotinamide adenine dinucleotide phosphate oxidase (NOX) is a key source of ROS in host cells. NOX is a multimer that requires all components for assembly and enzymatic activity in lipid rafts to produce ROS (49). Depletion of lipid rafts limits ROS production due to failure to recruit cytosolic components of the NOX complex (i.e., P47^phox^, P67^phox^, P40^phox^ and RAC) to the plasma membrane, which already harbors the gp91^phox^ and P22^phox^ components upon priming with interleukin 8 (Il-8) (50–52).Other adaptor molecules, such as protein kinase C involved in phosphorylating the NOX subunit P47^phox^, are also recruited to lipid rafts for ROS production. In another study, *Mycobacterium tuberculosis* 19-KDa lipoprotein, which is a TLR1/2 agonist, was shown to trigger translocation of TLR2 and protein kinase Cζ to lipid rafts and to induce ROS production (52). Disruption of lipid rafts in macrophages resulted in reduced *M. tuberculosis* lipoprotein-induced ROS production and recruitment of TLR2 and protein kinase Cζ, demonstrating that lipid rafts are critical to ROS production. Low doses of ROS under steady-state conditions contribute to cell survival, whereas high doses of ROS induced by infection help clear pathogens by activating cell death pathways such as apoptosis and necroptosis. In the TNF (tumor necrosis factor)-induced necroptosis pathway, activation of mixed lineage kinase domain-like protein (MLKL) lead to oligomerization of receptor-interacting protein3 (RIPK3) which is translocated to lipid rafts in the plasma membrane where it enhances sodium influx to induce cell rupture (53). Lipid rafts also regulate other cell death pathways such as autophagy. Lipid rafts are found in mitochondria associated membranes that connect ER with mitochondria and are required for the correct assembly of vesicles and formation of autophagosomes (54–56).

## Cellular Entry or Egress of Pathogens *via* Lipid Rafts

Host-pathogen interactions determine the outcome of infections. Lipid rafts are a key component of host-pathogen interactions on cell surfaces, given their roles in initiating cell signaling, harboring receptors and mediating cell trafficking (57–59). Experimental drugs that interrupt lipid raft formation have demonstrated that lipid rafts are important for cell entry of multiple viruses (**Table 1**). HIV-1 viral protein gp120 fuses with CD4^+^ T-cells through the lipid raft-associated receptors CD4, CCR5 and CXCR4 (60–62). Vaccinia mature virus (MV) clusters on lipid rafts where it interacts with the type II glycoprotein CD98 and integrin β1 to trigger endocytosis for cell entry (66, 67, 124). Both depletion of lipid rafts and knockdown of CD98 have been shown to reduce MV endocytosis, supporting the importance of lipid rafts in vaccinia virus entry into cells (66, 67). Hemagglutinin (HA) glycoprotein of influenza virus is important for virus-cell attachment and membrane fusion, which occurs more efficiently at lipid rafts of plasma membrane, suggesting that influenza virus employs lipid rafts for cell entry (69–71). Simian virus 40 (SV40), upon infection in cells, translocated to caveolae enriched membranes and specific disruption of caveolae with phorbol ester PMA or nystatin blocked SV40 entry (71). Human herpes virus-6 (HHV-6) enters cells through binding to cellular receptor CD46, which was enriched in lipid (74–77). As expected, depletion of cholesterol inhibited HHV-6 entry into cells (74–77). Similarly, poliovirus and type c foot-and mouth disease virus (FMDV) entry into the cells was also inhibited upon treatment with MβCD and was reversed upon addition of cholesterol suggesting role of lipid rafts in their entry (78, 79, 87). Flavivirus family members Japanese encephalitis virus (JEV), dengue virus serotype-2 (DEN-2) and West Nile virus (WNV) infection to cells was inhibited upon treatment with MβCD and cholesterol chelator filipin III. Surprisingly addition of cholesterol did not rescue cell susceptibility to JEV and DEN-2, unlike other viruses (81, 87). Several coronavirus family members such as mouse hepatitis virus (MHV), infectious bronchitis virus (IBV), human coronavirus 229E (HcoV-229E), severe acute respiratory syndrome virus (SARS-CoV), were shown to enter cells through lipid rafts (89, 91, 93, 96). A recent report also showed that pseudotyped virus containing SARS-CoV-2 spike protein enters cells through lipid rafts (99). African swine fever virus (ASFV) entry into pig macrophages is also dependent on lipid rafts as depletion of lipid rafts with cyclodextrins and nystatin blocked ASFV entry into pig macrophages (103, 104). Apart from viruses, bacterial pathogens also target lipid rafts during infection. For example, the enteric Gram-negative bacteria *Shigella flexneri* and *Salmonella enterica* enter cells by binding to lipid raft-associated receptors CD44 and CD55, respectively (125, 126). Depletion of lipid rafts impedes bacteria from binding to and entering host cells (127).

**Table 1:** Lipid raft mediated viral entry and receptors involved.

Virus	Receptors	Chemical and pharmaceutical drugs targeting lipid rafts	References
Human immunodeficiency virus	CD4, CCR5, CXCR4	MβCD, Cytochalasin, Nystatin, 25-Hydroxycholesterol, Atorvastatin	(60–65)
Vaccinia virus	CD98, Integrin β1	MβCD	(66–68)
Influenza	Sialic acid	MβCD, Cyclodextrin, Fluvastatin	(69–72)
Simian virus 40	Ganglioside GM1	PMA, Nystatin, Filipin III	(71, 73)
Human herpes virus-6	CD46	MβCD	(74–77)
Polio virus	CD155	MβCD	(78)
Foot-and-mouth disease virus	Integrin αvβ6	MβCD	(79, 80)
Japanese encephalitis virus	PLVAP, GKN3	MβCD, Filipin III	(81, 82)
Dengue virus	DC-SIGN, Mannose receptor, CLEC5A	MβCD, Filipin III, Cyclodextrins, Propofol,2,6-diisopropylphenol	(81, 83–86)
West Nile virus	TLR3	MβCD	(87, 88)
Mouse hepatitis virus	CD66a	MβCD, Filipin III	(89, 90)
Infectious bronchitis virus	Sialic acid	MβCD, Mevastatin	(91, 92)
Human coronavirus 229E	CD13	MβCD, Chloroquine	(93–95)
SARS-CoV	ACE2	MβCD, Cholesterol 25-Hydroxylase	(96–98)
SARS-CoV-2	ACE2	MβCD, Fluvoxamine, 25-Hydroxycholesterol, Fluvastatin	(98–102)
African swine fever virus	CD163	Cyclodextrins, Nystatin	(103, 104)
Zika virus	DC-SIGN, AXL, Tyro3, Tim-1	25-Hydroxycholesterol, Chloroquine	(83, 105, 106)
Hepatitis C virus	CD81, DC-SIGN, CD209L	Fluvastatin	(107, 108)
Respiratory Syncytial virus	CX3CR1, IGF1R	Lovastatin, Cyclodextrins	(83, 109–112)
Ebola virus	Tim-1	Lovastatin, cyclodextrins	(113, 114)
Herpes simplex virus	Heparan sulfate	Cyclodextrins	(83, 115)
Coxsackievirus	CAR	Fluoxetine	(116)
Enterovirus 71	SCARB2, Anx2, PSGL-1, sialylated glycan	Fluoxetine	(116–119)
Measles virus	CD46, SLAM	Halothane	(120, 121)
Murine Cytomegalovirus	Heparan sulfate	Simvastatin	(122, 123)

However, there are also studies indicating an opposing role for a lipid raft-associated protein, caveolin, during endocytic entry of the bacteria *Staphylococcus aureus* (128). Engagement of *S. aureus* by host integrin α5β1 *via* fibronectin was shown to trigger bacterial relocalization to lipid rafts. Surprisingly, caveolin deficiency, but not flotillin deficiency, enhanced *S. aureus* uptake. Recruitment of membrane lipids to the bacterial attachment site was not affected in Cav1^–/–^ cells, suggesting that caveolin blocks *S. aureus* in a post-attachment step (128). Caveolin has also been proven essential in host defenses against the pathogens *Pseudomonas aeruginosa* and *Salmonella enterica sv. Typhimurium* (129, 130). Caveolin-knockout mice are susceptible to both bacteria, exhibiting increased bacterial burdens in several organs relative to non-infected controls (129, 130). It is interesting that caveolin-knockout mice display a severe inflammatory phenotype, with elevated levels of inflammatory cytokines, chemokines in serum, and free radicals, implying complex biological functions of lipid raft-associated proteins in pathogen and host interactions (131). Pathogen interactions with lipid rafts are not solely restricted to the cell surface, since many intracellular pathogens could escape degradation by preventing phagosome fusion with lysosomes upon being internalized into cells (132). For example, *Leishmania donovani* utilizes lipophosphoglycans to disrupt dynein in lipid rafts and thereby avoids lysosomal degradation (132).

As for pathogen entry into cells, pathogen assembly and egress is crucial for its spread to other cells, tissues and hosts. Pathogens can manipulate the egress route by inducing programmed cell death (e.g., apoptosis) and cell rupture, by forming actin-mediated protrusions, or by inducing bud formation, with this latter potentially involving lipid rafts. Endosomal sorting complexes required for transport (ESCRT) are essential for membrane scission, as well as being involved in viral budding. Many enveloped viruses such as HIV, Murine Leukemia Virus (MLV) and vaccinia virus employ ESCRT-dependent budding or egress mechanisms (133–136). Knockdown of ESCRT complex members such as charged multivesicular body protein -2A (CHMP-2A) and CHMP-4B blocked the release of HIV and MLV (133, 136). Proteomic analyses have revealed the presence of ESCRT complex proteins in isolated lipid raft fractions, implying a role in viral budding (137, 138). Cholesterol synthesis inhibitor lovastatin treatment reduced the dengue virus (DENV) production by blocking virion assembly and intracellular trafficking showing that lipid rafts are important not only for DENV entry but also for their release from cells (139).

## Pharmaceutical Drugs Targeting Lipid Rafts to Block Pathogen Entry and Infection

As described above, many pathogen attachment receptors are concentrated in the lipid rafts, hence targeting lipid rafts could be a good strategy to overcome infection (140, 141). Disruption of lipid rafts by chemical compounds such as MβCD, filipin, 25-hydroxycholesterol and cyclodextrins has already shown to block the entry of many pathogens into cells (63, 81, 83, 100, 105, 142, 143). Furthermore, several pharmaceutical drugs used in treatment of other disorders were found to interact with lipid rafts resulting in blocking the entry of pathogens into the cells. Some of the well-known drugs targeting lipid rafts are statins (144, 145), anesthetics (146), and psychotropic drugs (147–150). Several statins such as lovastatin, mevastatin, fluvastatin, simvastatin, atorvastatin and nystatin used for the treatment of cardiovascular disease were reported to block virus entry into cells (64, 65, 72, 101, 107, 109, 110, 113, 122). Similarly, anesthetic drugs such as propofol, halothane and barbiturates were found to block virus entry due to their interaction with lipid rafts (84, 120). Antidepressants act by displacing G protein responsible for increasing cAMP (Gα_s_) from lipid rafts, and drugs such as fluvoxamine, and fluoxetine are known to block the entry of viruses (102, 116–118, 147, 149). Taken together, repurposing these existing drugs against newly emerging pathogenic virus may provide a good strategy since they have a proven safety record and can be deployed in a short time to treat viral infections. The list of potential pharmaceutical drugs targeting lipid rafts to block entry and propagation of viruses are included in **Table 1**.

## Major Vault Protein (MVP) Modulates Immune Signaling and Pathogen Entry

MVP is a 100-kDa protein that constitutes the major component of vault complex in cells (151, 152). The vault particle is a huge (400 Å x 670 Å) cage-like structure of 12.9 Mda, consisting of MVP, vault poly (ADP-ribose) polymerase (VPARP/PARP4) and telomerase associated protein (TEP1) proteins and multiple copies of small untranslated vault RNAs (vRNAs) (153). MVP is widely expressed in many normal tissues and overexpressed in many multi-drug-resistant cancer cells (153–155). MVP and vault particles may act as scaffolds for proteins involved in signal transduction, such as the Janus kinase/signal transducer and activator of transcription proteins (JAK/STAT) (156), Phosphoinositide-3-kinase/protein kinase B (PI3K/AKT) (125) and ERK (157) signaling pathways. MVP has also been implicated in suppression of metabolic diseases, such as obesity and atherosclerosis, through IKK-NF-κB signaling-mediated inflammation (158). MVP negatively regulates osteoclast differentiation by inhibiting the calcineurin-NFATc1 signaling pathway (159). Moreover, it interacts with Src in an epidermal growth factor (EGFR)-dependent manner and downregulates Src tyrosine kinase activity in stomach tissue, with this latter being necessary for activation of extracellular signal-regulated kinase (ERK) signaling (160).

**Figure 2:**
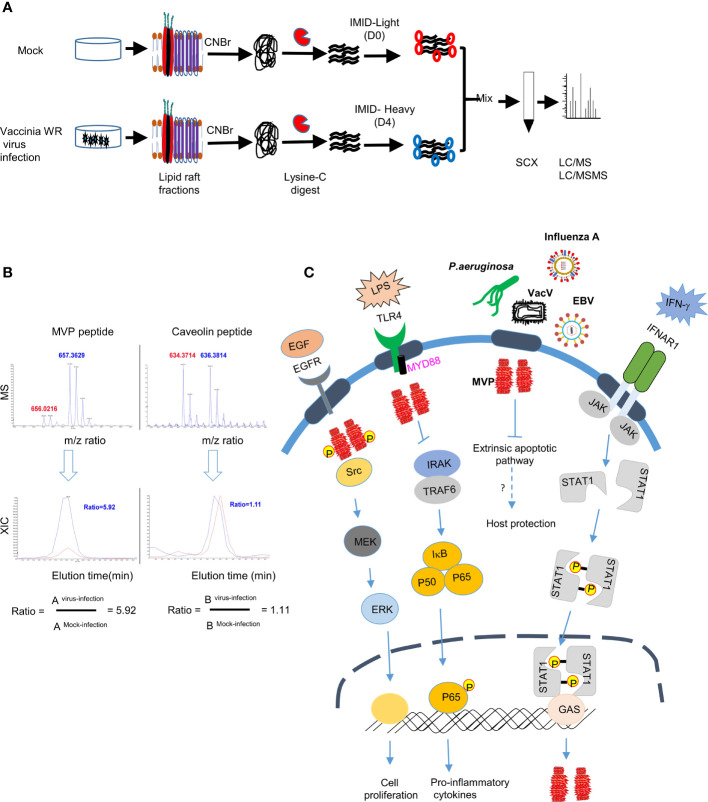
MVP accumulates in lipid rafts after infection with vaccinia virus **(A)** Schematic representation of differential IMID-H4/D4 labeling and LC/MS/MS analyses of lipid raft-associated proteins isolated from HeLa cells that were either mock infected or infected with WR strain MV as previously described (66, 67). (CNBr, Cyanogen bromide, SCX, Strong cation exchange column, m/z, mass-to-charge ratio), **(B)** Differential protein association in lipid raft proteome derived from mock or vaccinia virus infected HeLa cells (66). Labelled peptides were quantitatively determined by lysine-specific isotope labeling scheme. Co-eluted peaks contained peptides from both virus infected cells (blue) and from mock-infected cells (red). The blue-to-red ratio determined whether abundance of one particular peptide after virus infection is increased (>1), unchanged (=1) or decreased (<1). **(C)** Role of MVP in signaling pathways: MVP protein, the major component of vault particle, is recruited to the lipid rafts upon stimulation with growth factors (e.g., EGF) or pathogen derived ligands (e.g., LPS) and regulates important biological processes such as cell proliferation and cytokine secretion. IFNAR receptor stimulation with IFN-γ in the lipid rafts activates JAK-STAT signaling pathway which in turn will lead to the transcription of MVP. MVP also plays an important role in regulating apoptotic signaling pathway on infection with pathogens such as *P. aeruginosa* and helps in host protection, however the detailed mechanism on how recruitment of MVP to the lipid rafts after infection with other pathogens such as VacV, EBV and influenza virus needs investigation.

Interestingly, MVP and vault particles also play a role in pathogen, host interactions. For instance, vRNA induced by Epstein-Barr viral infections played a role in anti-viral host defense (161, 162). MVP was found to be enriched in lipid rafts following infection of human lung epithelial cells with *Pseudomonas aeruginosa* (163). Binding of *P. aeruginosa* LPS outer-core oligosaccharide to cystic fibrosis transmembrane conductance (CFTR) recruited MVP to lipid rafts and activate NF-κB signaling, IL-8 secretion and apoptosis induction. In MVP knockout (MVP^-/-^) mice bacteria uptake in lungs was reduced to 45% when compared with the wild-type mice. Further analyses concluded that MVP is critical for formation of stable membrane microdomains after *P. aeruginosa* infection. MVP translocation to lipid rafts was also induced by microbial metabolites such as N-(3-oxo-dodecanoyl) homoserine lactones (C12) released by proteobacteria and *Pseudomonas aeruginosa* (164) to modulate p38 pathway to reduce apoptotic cell death (164). In macrophages, MVP interacted with the scavenger receptor (SR-A/MSR1) in membrane rafts and modulated SR-A-caveolin-p38/JNK-mediated TNF-α production and apoptosis (165). MVP knockout (KO) mice grow normally showing that it is not required during mouse embryogenesis (166) and yet these KO mice are more susceptible to infection with several pathogens, such as Influenza A virus and *Pseudomonas aeruginosa*, suggesting that MVP plays an important role in immune responses against viral and bacterial pathogens (163, 167). In our previous study to identify cellular proteins enriched in lipid rafts upon vaccinia virus infection we identified integrin β1 and CD98 proteins that play important roles in virus entry (66, 67). Interestingly, these proteomic data (66) (**Figure 2A**) also revealed MVP to be enriched ~5-fold in lipid rafts upon vaccinia virus infection (**Figure 2B**) (66). Increase of MVP in lipid rafts is transient and MVP is not involved in integrin β1 or CD98 interactions and whether it participates in other signaling events during vaccinia mature virus entry remains to be investigated. The role of MVP in several immune signaling pathways, as summarized in (**Figure 2C**), showed that it may exert important functions in host-pathogen interactions, warranting further detailed experimental study.

## Conclusion and Future Perspectives

Lipid rafts on the plasma membrane are used as a portal for entry by many pathogens, including viruses and bacteria. Reorganization of cell surface lipid rafts during pathogen and virus entry could induce clustering of membrane receptors and/or intracellular molecules at the proximal inner membrane to facilitate entry, as well as to trigger signaling cascades. Thus, formation of such raft-associated protein complexes may reflect how pathogens and viruses engage with particular cellular receptors required for cell entry and invasion, how cells sense stress and mount immediate early anti-viral and anti-bacterial responses, and may even explain how viral proteins hijack lipid rafts to modify or antagonize cellular signaling and allow their propagation. Our understanding of the dynamic processes and kinetics governing lipid raft formation is still limited. This is in part due to technical challenges as well as the variety of different proteins found to be recruited to lipid rafts. Furthermore, one needs reliable and sensitive methods to modulate and monitor composition, molecular interactions and functionality in time, preferably in intact cells/tissues being challenged with pathogens. Reports in the literature highlight important, if not key roles for MVP recruited to lipid rafts upon pathogen exposure. Therefore, further detailed investigations are warranted to reveal the full functional potential of MVP in dealing with diverse pathogens in different cell types. A better and more precise delineation of the cellular function(s) of MVP, as well as the other components of the vault complex, will reveal interesting biology and possibly therapeutic opportunities.

## Author Contributions

RK, WC, and EW wrote and revised the manuscript. All authors contributed to the article and approved the submitted version.

## Funding

WC was supported by a grant from the Academia Sinica and Ministry of Science and Technology (110-2320-B-001-015-MY3).

## Conflict of Interest

The authors declare that the research was conducted in the absence of any commercial or financial relationships that could be construed as a potential conflict of interest.

## Publisher’s Note

All claims expressed in this article are solely those of the authors and do not necessarily represent those of their affiliated organizations, or those of the publisher, the editors and the reviewers. Any product that may be evaluated in this article, or claim that may be made by its manufacturer, is not guaranteed or endorsed by the publisher.

## Abbreviations

ACE2, Angiotensin converting enzyme 2; CLEC5A, C-type lectin 5A; DC-SIGN, Dendritic Cell-Specific Intercellular adhesion molecule-3-Grabbing Non-integrin; GKN3, Gastrokinase 3; PLVAP, Plasmalemma vesicle associated protein; PMA- Phorbol 12-myristate 13-acetate; IGF1R- Insulin like growth factor 1 receptor; Tim-1, T-cell immunoglobulin and mucin domain-1; CAR-Coxsakievirus and adenovirus receptor; SCARB2 - Scavenger receptor B2; PSGL-1 - P-selectin glycoprotein ligand-1; SLAM - Signaling lymphocytic activation molecule; Anx2 - Heparan sulfate and annexin II.
